# Risk factors of primary and recurrent fractures in postmenopausal osteoporotic Chinese patients: A retrospective analysis study

**DOI:** 10.1186/s12905-022-02034-z

**Published:** 2022-11-21

**Authors:** Xiaonan Zhu, Lin Chen, Ling Pan, Yuexi Zeng, Qiang Fu, Yanbin Liu, Yongde Peng, Yufan Wang, Li You

**Affiliations:** 1 Department of Endocrinology and Metabolism, Shanghai General Hospital, Shanghai Jiao Tong University School of Medicine, Shanghai, 200080 China; 2 Department of Orthopedics, Shanghai General Hospital, Shanghai Jiao Tong University School of Medicine, Shanghai, 200080 China

**Keywords:** Recurrent fractures, Primary fractures, Bone mineral density, Bone metabolism markers, Postmenopausal osteoporosis, Risk factors

## Abstract

**Background:**

As postmenopausal osteoporotic fractures can cause higher rates of disability and mortality in women; it is essential to analyze the factors associated with primary and recurrent fractures in postmenopausal osteoporosis (PMOP) patients.

**Methods:**

Retrospective analysis of 2478 PMOP patients aged ≥ 50 years who attended the Shanghai General Hospital from January 2007 to December 2016, including 1239 patients with no fractures and 1239 patients with histories of fractures (1008 in the primary fracture group and 231 in the re-fracture group). All patients' basic clinical data, serum biochemical and bone metabolic markers, bone mineral density (BMD), and other indicators were recorded uniformly. Comparing the differences between the clinical characteristics of patients with primary and recurrent fractures, as well as the differences in the clinical characteristics of patients with primary and recurrent fractures in combination with different diseases, further analyses the risk factors for primary and recurrent fractures in PMOP patients. SPSS.26 was used for statistical analysis.

**Results:**

Compared to the unfractured group, the fractured group was older and had lower height and bone mineral density (all *P* < 0.01), with the re-fractured group having lower BMD at each key site than the primary fracture group (all *P* < 0.01). Analysis of the combined disease subgroups showed that serum BGP levels were lower in the primary and re-fracture patients with diabetes than in the non-diabetic subgroup (*P *< 0.05), and serum CTX levels were lower in the re-fracture group with diabetes than in the primary fracture group with diabetes (*P* < 0.05). Patients with recurrent fractures with cardio-vascular diseases had lower BMD than the subgroup without cardio-vascular diseases (*P* < 0.05) and also had lower BMD than the group with primary fractures with cardio-vascular diseases (*P* < 0.05). Multiple logistic regression analysis showed that advanced age, overweight, low lumbar spine and total hip BMD were risk factors for primary and recurrent fractures; and comorbid chronic liver and kidney diseases were risk factors for primary fractures.

**Conclusion:**

PMOP patients with advanced age, overweight, low bone mineral density, and comorbid chronic liver and kidney diseases are at greater risk of fractures and require early intervention to reduce fractures occurrence. Moreover, those who are elderly, overweight, and have low bone density should also be aware of the risk of re-fractures.

## Introduction

Osteoporosis is a systemic bone disease characterized by low bone mass, damage to the microstructure of bone tissue and increased bone fragility [1, 2]. Osteoporotic fractures, also known as fragility fractures, are fractures that occur with minor trauma or during everyday activities and can lead to high rates of disability and death, particularly hip and vertebral fractures [1, 2]. Postmenopausal women are prone to osteoporosis 5–10 years after menopause due to estrogen deficiency and are more likely than men to develop osteoporotic fractures earlier [1, 3]. In addition, the financial burden of osteoporotic fractures on patients and health care systems is significant. Therefore, the prevention and treatment of osteoporotic fractures are of great importance. In the early days, scholars focused on the factors associated with primary fractures in postmenopausal osteoporosis (PMOP) patients, these studies showed that bone mineral density and bone metabolic markers levels were significantly associated with primary fractures in PMOP patients, and advanced age, body mass index, cognitive ability, diabetes, falls, and physical function were also predictive factors of primary fractures [4–7]. In recent years, it has been recognized that PMOP patients are also at high risk of recurrent fractures after the primary fractures. Balasubramanian [8], Bliuc [9] et al. showed that postmenopausal women have a further increased risk of recurrent fractures within 5 years of the primary fractures and that recurrent fractures significantly increase the overall mortality associated with fractures. However, at the same time, we have observed from clinical practice that not all patients with primary osteoporotic fractures will experience recurrent fractures. It is suggested that some risk factors that may lead to recurrent fractures require need special attention and a comprehensive and systematic study of the risk factors for recurrent fractures is really necessary. Although a number of scholars have investigated the risk factors associated with re-fracture in PMOP patients, they have mainly focused on the effect of treatments of the initial fractures on recurrent fractures [10, 11] and the correlation between the sites of initial fractures and re-fractures [12], etc. However, there are fewer comprehensive retrospective analyses of the impact of baseline level clinical characteristics and co-morbidities on initial and recurrent fractures in patients with PMOP. In this study, we aimed to investigate the risk factors associated with patients with primary and recurrent fractures, to provide some theoretical basis for effective prevention of primary and recurrent fractures in PMOP patients.

## Methods and materials

### Participants and study design

**Fig.1:**
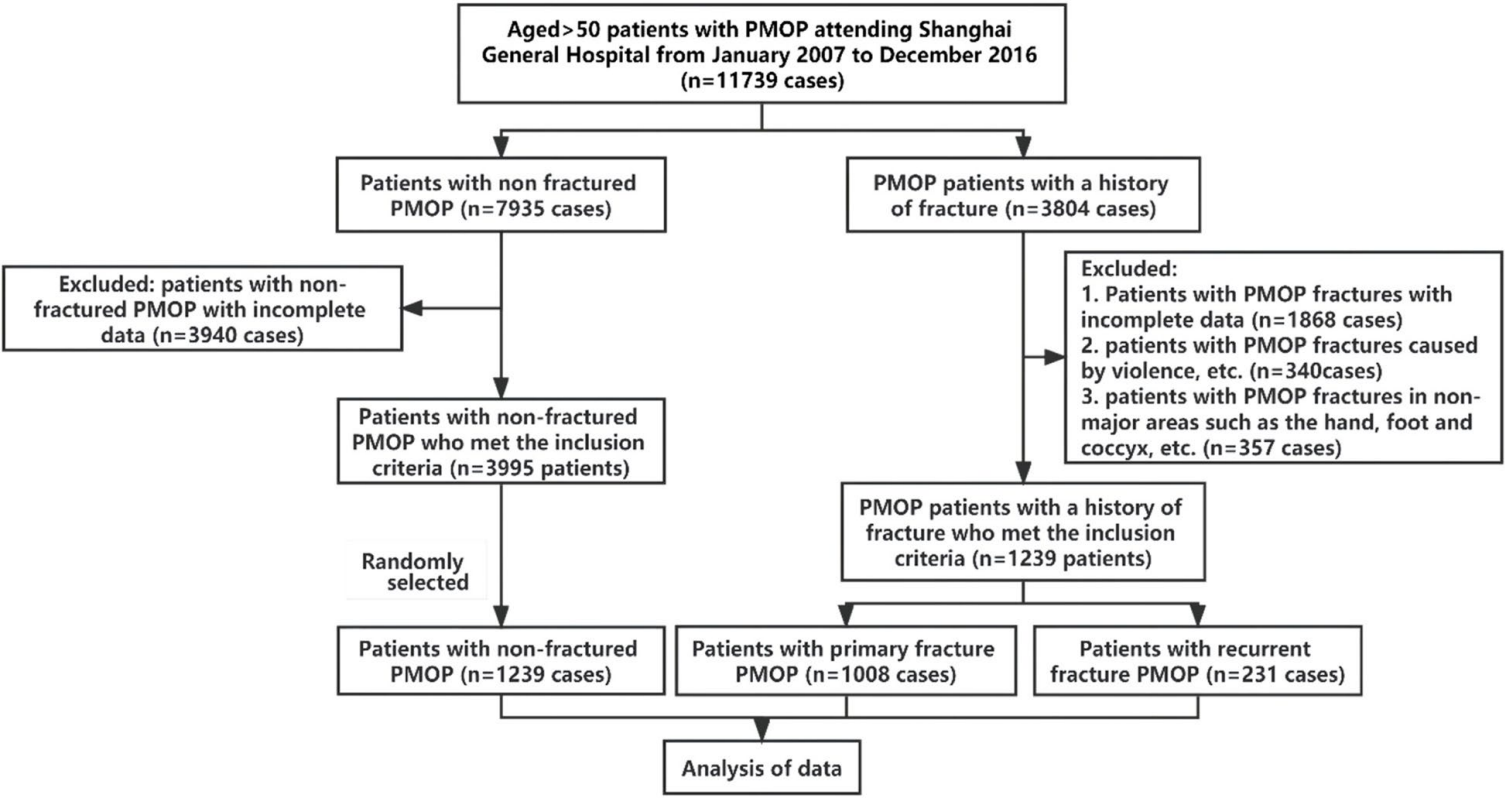
Flowchart of the patient-selection process

In this study, 11,739 PMOP patients aged ≥ 50 who attended Shanghai General Hospital from January 2007 to December 2016 were selected, including 7935 PMOP patients without fracture and 3,804 PMOP patients with histories of fracture. We used the “EPV (events per variable)” empirical method to estimate the sample size. Sixteen independent variables were included in the study: age, weight, height, L1-4 BMD, femoral neck BMD, total hip BMD, serum Ca, P, PTH, 25OHD, BGP, CTX, with or without diabetes, with or without chronic gastric diseases, with or without Cardio-cerebrovascular diseases, and with or without chronic liver or kidney diseases. With our assumption of EPV = 15, the number of fracture cases required was 16 × 15 = 240. And as it is known from previous literature that the incidence of postmenopausal osteoporotic fractures is 44.3% [13], the total sample size is 240/44.3% = 558 cases, i.e., the sample size of the non-fractured group is 318 cases. After setting the inclusion and exclusion criteria, we statistically analyzed the data of patients who met the inclusion criteria, including 1239 randomly selected cases from the unfractured patients who met the inclusion criteria, and 1239 patients with fractures (1008 patients with primary fractures and 231 patients with re-fractures) (Fig. 1).

#### Inclusion criteria

All study subjects were postmenopausal women aged ≥ 50 years with osteoporosis. Post-menopausal women were those who had not menstruated at least 1 year due to loss of ovarian follicular activity [14]. The diagnosis of osteoporosis is based on the diagnostic criteria of the China 2017 guidelines for the management of primary osteoporosis and the American College of Endocrinology 2020 clinical practice guidelines for the diagnosis and treatment of postmenopausal osteoporosis [1, 2]. Diagnosis of osteoporotic fracture is based on a self-reported history of non-violent fracture or imaging suggestive of fracture of vertebrae, hip, etc. and a BMD T value < -2.5 SD measured by DXA (dual energy x-ray absorptiometry). Self-reported fractures (including primary fractures and re-fractures) are mainly those resulting from non-violent factors such as daily activities or minor trauma and supported by clinical records such as (operation reports, clinical records, previous or current X-rays). Fracture sites included major fracture sites: vertebrae, hip, pelvis, proximal humerus, distal forearm, and ankle; all patients signed an informed consent form. Exclusion criteria: fractures due to violence such as those caused by car accidents, falls and high-energy impacts; pathological fractures due to bone metastases from malignant tumors; fractures of non-major parts of the body such as the hand, foot, and tailbone; Secondary osteoporosis and other metabolic bone diseases caused by a history of drug use, steroid and Vit D use, etc.

In this study, general clinical information, including age, height, weight, body mass index (BMI), bone mineral density and bone metabolic markers, as well as disease history, were collected from all participants uniformly at the time of the patients' outpatient visits to the Shanghai General Hospital. we retrospective comparative analysis of the differences in the baseline BMD and bone metabolic markers between the unfractured group, the primary fracture group and the re-fracture group, as well as the differences in BMD and bone metabolic markers between primary and recurrent fracture patients with combined diabetes mellitus, Cardio-cerebrovascular diseases, chronic liver and kidney diseases and chronic gastric diseases. Regression analysis was performed to determine the risk factors for primary and recurrent fractures in patients with PMOP.

### Data collection

In this study, general information was measured and collected uniformly from the study subjects. The height and weight of the enrolled patients were measured by a height and weight measuring device (Omron, Japan), and the corresponding BMI was calculated. Their age, comorbidities, and specific fracture sites were also recorded.

#### Bone metabolic markers and biochemical tests

Early morning fasting venous blood was drawn from 8:00 am-10:00 am from the enrolled patients and stored in a refrigerator (Haier, China) at -80℃. The biochemical markers are measured by electrochemiluminescence (Roche E170, Germany) and the specific reagents are from Roche Diagnostics GmbH, Germany. The main indicators measured were serum bone Gla protein (BGP), C-terminal telopeptide of type 1 collagen (CTX), calcium (Ca), phosphorus (P), parathyroid hormone (PTH), and25 hydroxyvitamin D (25 OHD).

#### Bone mineral density (BMD) testing

BMD of orthostatic lumbar L1-4, left femoral neck, and left total hip was measured using a dual-energy X-ray BMD machine (GE, USA). Body mold tests were performed before measurement. The coefficients of variation (CV) values were 1.4% for L1-4, 1.2% for the total hip, and 1.7% for the femoral neck. And each measurement was subjected to strict quality control.

### Statistical analysis

All analyses were performed using IBM SPSS Statistics version 26.0. We used one-way ANOVA for measurement data and the Bonferroni, Dunnett3 methods for post-hoc comparisons, with results expressed as mean ± SD values. The Kendall correlation was used to analyze the correlation between fracture history and clinical indicators. Multivariate logistic regression analysis was performed to analyze the risk factors for primary and recurrent fractures in patients with PMOP. P-values less than 0.05 were considered the statistically significant differences.

## Results

### Percentage of different fracture sites in patients with primary and recurrent fractures.

**Fig. 2:**
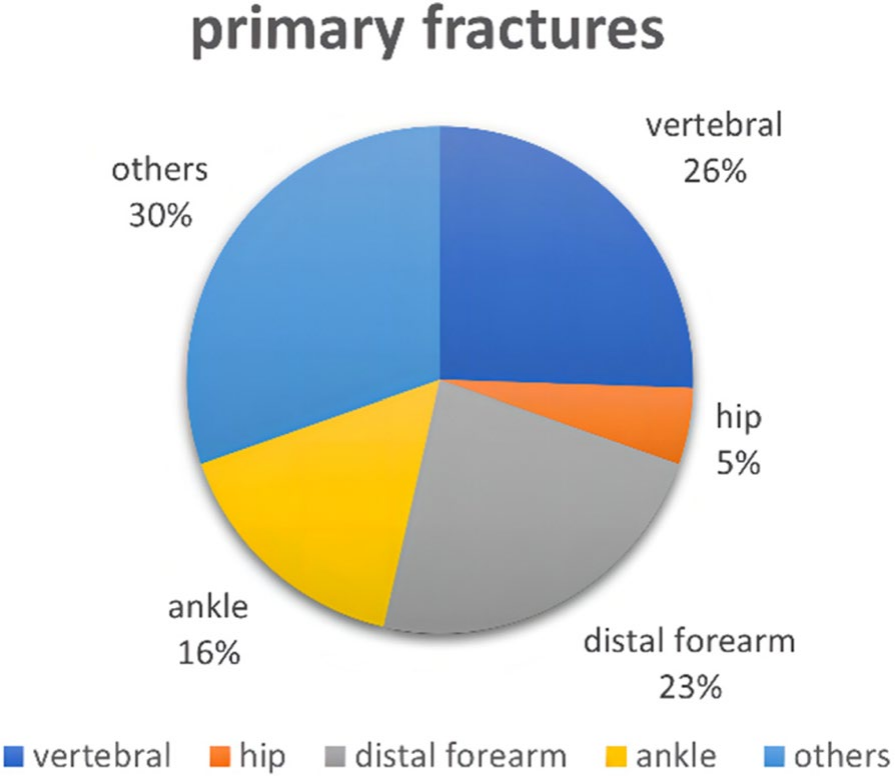
Percentage of different fracture sites in patients with primary fractures

**Fig. 3:**
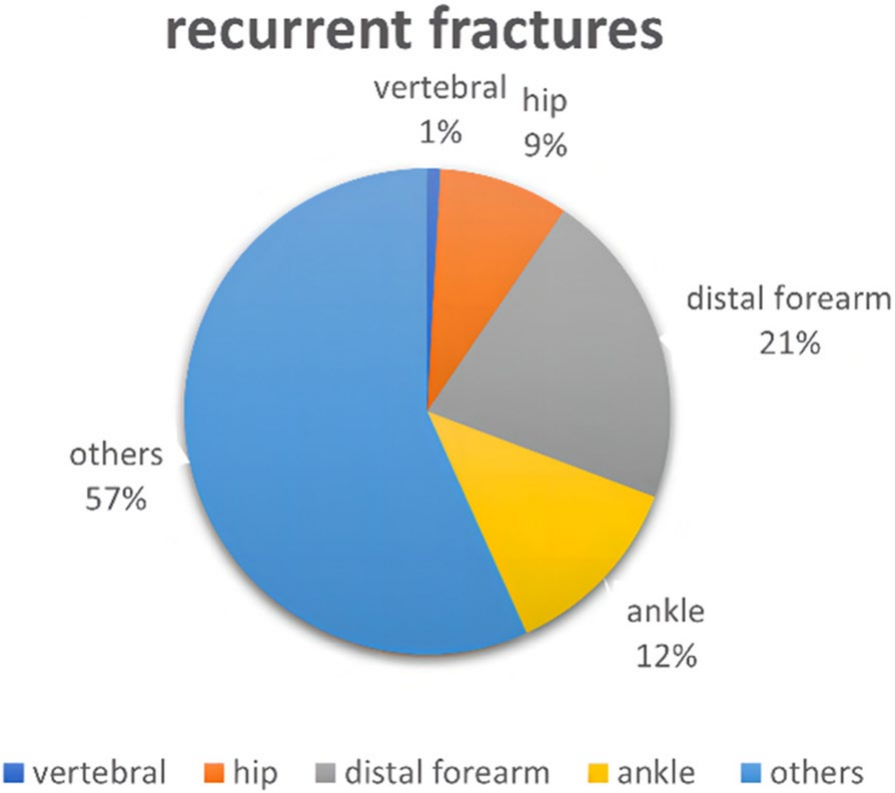
Percentage of different fracture sites in patients with recurrent fractures

Among patients with primary fractures: 26% of vertebral fractures, 5% of hip fractures, 16% of ankle fractures, 23% of distal forearm fractures and 30% of fractures of other sites. Among patients with recurrent fractures: vertebral fractures of 1%, hip fractures of 9%, ankle fractures of 12%, distal forearm fractures of 21% and fractures of other sites of 57% (Figs. 2 and 3).

### Comparison of basic clinical information and bone mineral density and bone metabolic markers among the groups

Compared with the non-fracture group the age was higher (*P* < 0.01) and the height was lower (both *P* < 0.01) in the primary and re-fractured groups; the weight was lower (*P* < 0.05) in the primary fracture group; the weight was lower in the re-fractured group than in the non-fractured group, but the difference was not statistically significant (*P* > 0.05) (Table 1).

**Table 1:** Basic clinical information of patients among the groups

Groups	Non-fracture group (*n* = 1239)	Primary fracture group (*n* = 1008)	Re-fracture group (*n* = 231)
Age (year)	64.343 ± 9.109	69.905 ± 9.757**	71.070 ± 10.179**
Height (cm)	157.917 ± 5.743	156.702 ± 6.123**	155.866 ± 6.224**
Weight (kg)	58.018 ± 8.747	57.075 ± 9.134*	56.974 ± 9.873
BMI (kg/m^2^)	23.299 ± 3.503	23.213 ± 3.369	23.447 ± 3.854

Compared with the non-fracture group: * indicates *P* < 0.05, ** indicates *P* < 0.01; *BMI* body mass index

Compared with the non-fracture group, L1-4 BMD, femoral neck BMD and total hip BMD were significantly lower in both the primary and re-fracture groups (all *P* < 0.01), except for the lower serum P level in the primary fracture group compared with the non-fracture group (*P* < 0.01), there were no statistical differences between the bone metabolic markers in the other groups. L1-4 BMD, femoral neck BMD, and total hip BMD were significantly lower in the re-fracture group compared to the primary fracture group (all *P* < 0.01) (Table 2).

**Table 2:** Comparison of BMD and bone metabolic markers of patients among groups

Groups	Non-fracture group (*n* = 1239)	Primary fracture group (*n* = 1008)	Re-fracture group (*n* = 231)
L1-4BMD (g/cm^2^)	0.992 ± 0.169	0.908 ± 0.163**	0.858 ± 0.149**®
Total hip BMD (g/cm^2^)	0.837 ± 0.138	0.757 ± 0.134**	0.715 ± 0.133**®
Femoral neck BMD (g/cm^2^)	0.770 ± 0.138	0.701 ± 0.119**	0.667 ± 0.115**®
Serum BGP (ng/mL)	21.048 ± 16.723	20.821 ± 13.330	19.670 ± 8.321
Serum CTX (pg/mL)	474.510 ± 280.312	471.710 ± 249.419	464.598 ± 222.905
Serum Ca (mmol/L)	2.280 ± 0.127	2.280 ± 0.122	2.272 ± 0.126
Serum P (mmol/L)	1.221 ± 0.177	1.195 ± 0.169**	1.196 ± 0.155
Serum PTH (pg/mL)	44.515 ± 27.545	46.151 ± 23.154	46.500 ± 22.257
Serum 25OHD (nmol/L)	52.401 ± 19.709	54.037 ± 36.010	51.780 ± 20.102

Compared to the non-fracture group: * indicates *P* < 0.05 and ** indicates *P* < 0.01; compared to the primary fracture group, ®indicates *P* < 0.01

### Comparison of bone mineral density and bone metabolic markers in PMOP primary fracture patients with different comorbidities

As shown in Table 3, in the primary fracture group: L1-4 BMD was lower in the group with the chronic gastric disease than in the group without the chronic gastric diseases (*P* < 0.05). The serum BGP level was lower in the group with diabetes than in the group without diabetes (*P* < 0.05); there was no difference between all other groups in all indicators (*P* > 0.05).

**Table 3:** Comparison of clinical characteristics of patients with primary fractures with different comorbidities

Groups	Diabetes			Cardio-cerebrovascular diseases			Chronic liver and kidney diseases			Chronic gastric diseases		
	Yes (*n* = 103)	No (*n* = 905)	P	Yes (*n* = 230)	No (*n* = 778)	P	Yes (*n* = 89)	No (*n* = 919)	P	Yes (*n* = 61)	No (*n* = 947)	P
L1-4BMD (g/cm^2^)	0.919 ± 0.156	0.906 ± 0.164	0.462	0.915 ± 0.160	0.905 ± 0.165	0.425	0.939 ± 0.148	0.905 ± 0.165	0.054	0.866 ± 0.129	0.910 ± 0.165	0.014*
Total hip BMD (g/cm^2^)	0.767 ± 0.129	0.755 ± 0.134	0.410	0.744 ± 0.122	0.760 ± 0.137	0.083	0.762 ± 0.114	0.756 ± 0.136	0.621	0.748 ± 0.100	0.757 ± 0.136	0.510
Femoral neck BMD (g/cm^2^)	0.697 ± 0.118	0.710 ± 0.120	0.754	0.689 ± 0.111	0.704 ± 0.122	0.087	0.713 ± 0.101	0.699 ± 0.121	0.241	0.697 ± 0.088	0.701 ± 0.121	0.767
Serum BGP (ng/mL)	17.732 ± 8.914	21.171 ± 13.710	0.013*	21.111 ± 10.082	20.734 ± 14.161	0.706	24.043 ± 31.639	20.508 ± 9.902	0.297	20.911 ± 6.464	20.814 ± 13.663	0.956
Serum CTX (pg/mL)	466.968 ± 251.334	472.250 ± 249.335	0.839	498.15 ± 286.683	463.893 ± 236.927	0.099	517.143 ± 341.965	467.310 ± 238.352	0.182	463.267 ± 216.814	472.254 ± 251.466	0.785
Serum Ca (mmol/L)	2.266 ± 0.110	2.282 ± 0.123	0.224	2.293 ± 0.125	2.277 ± 0.121	0.080	2.285 ± 0.139	2.280 ± 0.120	0.713	2.301 ± 0.149	2.279 ± 0.120	0.173
Serum P (mmol/L)	1.999 ± 0.201	1.194 ± 0.166	0.783	1.196 ± 0.205	1.194 ± 0.157	0.906	1.171 ± 0.166	1.197 ± 0.170	0.171	1.203 ± 0.186	1.194 ± 0.168	0.670
Serum PTH (pg/mL)	45.342 ± 18.048	46.243 ± 23.672	0.708	47.853 ± 24.717	45.648 ± 22.663	0.205	47.008 ± 27.107	46.068 ± 22.750	0.715	44.073 ± 21.390	46.285 ± 23.267	0.470
Serum 25OHD (nmol/L)	53.178 ± 40.188	54.135 ± 35.525	0.799	54.176 ± 39.733	53.996 ± 34.859	0.947	54.833 ± 36.814	53.960 ± 35.950	0.827	58.642 ± 42.079	53.740 ± 35.588	0.303

^*^ indicates *P* < 0.05

### Comparison of bone mineral density and bone metabolic markers in patients with PMOP re-fractures in combination with different diseases

As shown in Table 4, among the re-fracture groups: serum BGP and serum CTX levels were significantly lower in the group with diabetes than in the group without diabetes (*P* < 0.05); total hip BMD and femoral neck BMD were lower in the group with cardio-cerebrovascular diseases than in the group without the cardio-cerebrovascular diseases (*P* < 0.05); there were no statistical differences between the indicators in the remaining groups (*P* > 0.05).

**Table 4:** Comparison of clinical characteristics of patients with re-fractures with different comorbidities

Groups	Diabetes			Cardio-cerebrovascular diseases			Chronic liver and kidney diseases			Chronic gastric diseases		
	Yes (*n* = 30)	No (*n* = 201)	P	Yes (*n* = 60)	No (*n* = 171)	P	Yes (*n* = 16)	No (*n* = 215)	P	Yes (*n* = 13)	No (*n* = 218)	P
L1-4BMD (g/cm^2^)	0.893 ± 0.144	0.852 ± 0.149	0.166	0.836 ± 0.134	0.865 ± 0.154	0.188	0.881 ± 0.170	0.856 ± 0.148	0.523	0.850 ± 0.147	0.858 ± 0.149	0.845
Total hip BMD (g/cm^2^)	0.734 ± 0.123	0.712 ± 0.134	0.412	0.680 ± 0.131	0.728 ± 0.132	0.016*	0.724 ± 0.100	0.714 ± 0.135	0.789	0.679 ± 0.099	0.717 ± 0.135	0.317
Femoral neck BMD (g/cm^2^)	0.684 ± 0.101	0.664 ± 0.117	0.360	0.632 ± 0.112	0.679 ± 0.115	0.006*	0.662 ± 0.092	0.667 ± 0.117	0.869	0.621 ± 0.067	0.669 ± 0.117	0.140
Serum BGP (ng/mL)	14.753 ± 6.202	20.416 ± 8.340	0.000*	20.892 ± 10.384	19.248 ± 7.406	0.185	20.671 ± 4.750	19.608 ± 8.513	0.622	18.149 ± 5.735	19.773 ± 8.434	0.495
Serum CTX (pg/mL)	344.693 ± 209.605	482.494 ± 219.751	0.001*	472.053 ± 224.535	461.922 ± 222.922	0.761	529.319 ± 254.836	459.781 ± 220.257	0.229	379.754 ± 168.659	469.657 ± 225.035	0.158
Serum Ca (mmol/L)	2.281 ± 0.108	2.271 ± 0.129	0.699	2.282 ± 0.164	2.269 ± 0.110	0.571	2.287 ± 0.135	2.271 ± 0.126	0.634	2.235 ± 0.101	2.275 ± 0.127	0.268
Serum P (mmol/L)	1.225 ± 0.181	1.192 ± 0.151	0.280	1.174 ± 0.135	1.204 ± 0.162	0.189	1.251 ± 0.130	1.192 ± 0.157	0.148	1.165 ± 0.178	1.198 ± 0.154	0.449
Serum PTH (pg/mL)	42.115 ± 19.887	47.155 ± 22.561	0.248	49.945 ± 31.054	45.264 ± 18.055	0.270	44.888 ± 22.013	46.620 ± 22.321	0.765	52.269 ± 17.064	46.156 ± 22.513	0.337
Serum 25OHD (nmol/L)	53.134 ± 19.276	51.429 ± 20.246	0.493	52.884 ± 22.657	51.384 ± 19.160	0.618	63.004 ± 29.941	50.945 ± 19.010	0.132	44.052 ± 23.617	52.241 ± 19.841	0.154

^*^ indicates *P* < 0.05

### Comparison of BMD and bone metabolic markers between patients with primary and recurrent fractures of PMOP combined with different diseases

As shown in Table 5, compared with the primary fracture group: serum CTX levels were lower in the re-fracture group with combined diabetes (*P* < 0.05); L1-4 BMD, femoral neck BMD, and total hip BMD levels were lower in the re-fracture group with combined cardio-cerebrovascular diseases (*P* < 0.05); femoral neck BMD and total hip BMD levels were lower in the re-fracture group with combined chronic gastric diseases (*P* < 0.05) The rest of the bone metabolic markers were not statistically different between the groups (*P* > 0.05).

**Table 5:** Comparison of clinical characteristics of patients with primary and re-fractures with different comorbidities

Groups	Diabetes			Cardio-cerebrovascular diseases			Chronic liver and kidney diseases			Chronic gastric diseases		
	Primary (*n* = 103)	Recurrent (*n* = 30)	P	Primary (*n* = 230)	Recurrent (*n* = 60)	P	Primary (*n* = 89)	Recurrent (*n* = 16)	P	Primary (*n* = 61)	Recurrent (*n* = 13)	P
L1-4BMD (g/cm^2^)	0.919 ± 0.156	0.893 ± 0.144	0.412	0.915 ± 0.160	0.836 ± 0.134	0.000*	0.939 ± 0.148	0.881 ± 0.170	0.154	0.866 ± 0.129	0.850 ± 0.147	0.680
Total hip BMD (g/cm^2^)	0.767 ± 0.129	0.734 ± 0.123	0.214	0.744 ± 0.122	0.680 ± 0.131	0.000*	0.762 ± 0.114	0.724 ± 0.100	0.204	0.748 ± 0.100	0.679 ± 0.099	0.027*
Femoral neck BMD (g/cm^2^)	0.697 ± 0.118	0.684 ± 0.101	0.605	0.689 ± 0.111	0.632 ± 0.112	0.000*	0.713 ± 0.101	0.662 ± 0.092	0.064	0.697 ± 0.088	0.621 ± 0.067	0.004*
Serum BGP (ng/mL)	17.732 ± 8.914	14.753 ± 6.202	0.207	21.111 ± 10.082	20.892 ± 10.384	0.881	24.043 ± 31.639	20.671 ± 4.750	0.673	20.911 ± 6.464	18.149 ± 5.735	0.159
Serum CTX (pg/mL)	466.968 ± 251.334	344.693 ± 209.605	0.017*	498.153 ± 286.683	472.053 ± 224.535	0.510	517.143 ± 341.965	529.319 ± 254.836	0.892	463.267 ± 216.814	379.754 ± 168.659	0.196
Serum Ca (mmol/L)	2.266 ± 0.110	2.281 ± 0.108	0.530	2.293 ± 0.125	2.282 ± 0.164	0.636	2.285 ± 0.139	2.287 ± 0.135	0.954	2.301 ± 0.149	2.235 ± 0.101	0.132
Serum P (mmol/L)	1.999 ± 0.201	1.225 ± 0.181	0.522	1.196 ± 0.205	1.174 ± 0.135	0.435	1.171 ± 0.166	1.251 ± 0.130	0.071	1.203 ± 0.186	1.165 ± 0.178	0.493
Serum PTH (pg/mL)	45.342 ± 18.048	42.115 ± 19.887	0.401	47.853 ± 24.717	49.945 ± 31.054	0.579	47.008 ± 27.107	44.888 ± 22.013	0.768	44.073 ± 21.390	52.269 ± 17.064	0.200
Serum 25OHD (nmol/L)	53.178 ± 40.188	53.134 ± 19.276	0.900	54.176 ± 39.733	52.884 ± 22.657	0.908	54.833 ± 36.814	63.004 ± 29.941	0.404	58.642 ± 42.079	44.052 ± 23.617	0.232

^*^indicates *P* < 0.05

### Correlation analysis between fracture history and various indicators

As shown in Table 6, history of fracture was positively correlated with age (*r* = 0.231, *P* = 0.000), PTH (*r* = 0.039, *P* = 0.014), diabetes (*r* = 0.044, *P* = 0.024), Cardio-cerebrovascular diseases (*r* = 0.105, *P* = 0.000) and chronic liver and kidney diseases (*r* = 0.043, *P* = 0.027). History of fracture was negatively correlated with height (*r* = -0.089, *P* = 0.000), weight (*r* = -0.041, *P* = 0.011), L1-4 BMD (*r* = -0.221, *P* = 0.000), femoral neck BMD (*r* = -0.244, *P* = 0.000), total hip BMD (*r* = -0.256, *P* = 0.000), serum P (*r* =-0.059, *P* = 0.000), while there was no correlation with the remaining indicators.

**Table 6:** Correlation between fracture history and indicators (r/P)

Indicators	r	P
Age (year)	0.231	0.000*
Height (cm)	-0.089	0.000*
Weight (kg)	-0.041	0.011*
BMI (kg/m^2^)	0.006	0.714
Serum Ca (mmol/L)	-0.015	0.352
Serum P (mmol/L)	-0.059	0.000*
Serum PTH (pg/mL)	0.039	0.014*
Serum 25OHD (nmol/L)	-0.017	0.272
Serum BGP (ng/mL)	-0.006	0.723
Serum CTX (pg/mL)	-0.002	0.911
L1-4 BMD (g/cm^2^)	-0.221	0.000*
Total hip BMD (g/cm^2^)	-0.244	0.000*
Femoral neck BMD (g/cm^2^)	-0.256	0.000*
Diabetes	0.044	0.024*
Cardio-cerebrovascular diseases	0.105	0.000*
Chronic gastric diseases	0.008	0.665
Chronic liver and kidney diseases	0.043	0.027*

^*^ indicates *P* < 0.05

### Multiple logistic regression analysis of fracture history

As shown in Table 7, the history of primary fracture and history of re-fracture were used as dependent variables, and the non-fracture group was the reference group. All factors with P < 0.15 in the above correlation analysis (age, weight, height, L1-4 BMD, femoral neck BMD, total hip BMD, serum P, serum PTH and comorbidities) were further used as independent variables in a multivariate logistic regression analysis.

**Table 7:** Multivariate logistic regression analysis of fracture history in PMOP patients

Groups	Primary fractures		Recurrent fractures	
	Odds Ratio (95%CI)	P	Odds Ratio (95%CI)	P
Age (year)	1.047 (1.035–1.059)	0.000	1.039 (1.026–1.059)	0.000
Weight (kg)	1.019 (1.007–1.031)	0.002	1.050 (1.020–1.070)	0.000
Height (cm)	1.011 (0.993–1.030)	0.220	0.989 (0.961–1.018)	0.472
L1-4BMD (g/cm^2^)	0.119 (0.058–0.243)	0.000	0.022 (0.007–0.076)	0.000
Femoral neck BMD (g/cm^2^)	2.745 (0.503–14.985)	0.244	5.608 (0.395–79.675)	0.203
Total hip BMD (g/cm^2^)	0.085 (0.016–0.462)	0.004	0.006 (0.000–0.089)	0.000
Serum P (mmol/L)	0.683 (0.405–1.153)	0.153	0.935 (0.395–2.211)	0.878
Serum PTH (pg/mL)	1.000 (0.996–1.003)	0.977	1.000 (0.994–1.006)	0.941
Diabetes	1.041 (0.766–1.415)	0.798	1.327 (0.834–2.110)	0.232
Cardio-cerebrovascular diseases	1.124 (0.891–1.418)	0.325	1.306 (0.914–1.865)	0.142
Chronic liver and kidney diseases	1.415 (1.007–1.988)	0.046	1.123 (0.627–2.011)	0.696

It can be concluded that age (OR = 1.047, *P* = 0.000), weight (OR = 1.019, *P* = 0.002) and chronic liver and kidney diseases (OR = 1.415, *P* = 0.046) were risk factors for primary fractures in patients with PMOP; L1-4 BMD (OR = 0.119, *P* = 0.000) and total hip (OR = 0.085, *P* = 0.004) were protective factors for primary fractures. Risk factors for recurrent fractures in PMOP patients included age (OR = 1.039, *P* = 0.000), weight (OR = 1.050, *P* = 0.000); L1-4 BMD (OR = 0.022, *P* = 0.000), total hip BMD (OR = 0.006, *P* = 0.000) were protective factors for recurrent fractures.

## Discussion

Postmenopausal women are at increased risk of fragility fractures due to estrogen deficiency, which causes accelerated bone loss and damage to bone tissue microstructure. Studies have shown that 1 in 2 postmenopausal women with a recent fracture will have a recurrent fracture within 5 years [15]. The occurrence of recurrent fractures leads to increased disability and mortality in postmenopausal women with osteoporosis. Therefore, early interventions to identify risk factors for both primary and recurrent fractures are essential to prevent recurrent fractures in postmenopausal women at risk for osteoporotic fractures. In this study, we systematically compared the clinical characteristics (basic data and co-morbidities) and bone metabolism of patients with primary and recurrent fractures in PMOP. The regression analysis showed that advanced age, overweight, low L1-4BMD and total hip BMD, and combined chronic liver and kidney diseases were risk factors for primary fracture in PMOP patients, and advanced age, overweight, low L1-4BMD and total hip BMD were also risk factors for recurrent fractures in PMOP patients.

Postmenopausal women have increased osteoclast activity due to estrogen deficiency, and enhanced oxidative stress in the skeleton with age also inhibits osteoclast formation, resulting in accelerated bone loss [4, 16]. In this study, the age of fractured patients was higher than that of non-fracture patients, and age was positively correlated with fracture history. Regression analysis showed that advanced age was an independent risk factor for primary and recurrent fractures in PMOP patients, which is consistent with the results of Hadji [17] and Zhuang [18] et al. This suggests that the risk of primary and recurrent osteoporotic fractures increases with age in patients with PMOP.

The relationship between body weight and osteoporotic fractures is currently controversial. Early studies suggested that low body weight is a risk factor for osteoporotic fractures [19, 20]. However, several recent studies have shown that the effect of obesity on osteoporotic fractures is related to the sites of fractures. Obese women have a higher incidence of vertebral, ankle and humeral fractures and a lower incidence of hip fractures compared to non-obese women [21, 22]. Also, Tanaka S [23] et al. Found overweight, obesity and underweight to be risk factors for fractures at different sites respectively. In this study, overweight was a risk factor for both primary and recurrent fractures in PMOP patients, which may be because hip fractures account for a smaller proportion of primary and recurrent fractures in PMOP patients, and fractures at sites such as the vertebrae and ankle and distal forearm are more prevalent (Figs. 2 and 3). Studies have shown that obesity may reduce bone strength by affecting the bone material composition and that increased adipose tissue within the muscle may lead to increased muscle weakness and risk of falls, making the increased risk of fractures [24].

BMD is not only a risk assessment tool for osteoporotic fractures but also a risk factor for osteoporotic fractures. In the present study, BMD was lower in both the PMOP primary fracture group and the re-fracture group than in the non-fracture group. Moreover, the BMD in the re-fracture group was lower than that in the primary fracture group. Further regression analysis in this study showed that low L1-4 BMD and total hip BMD were independent risk factors for both primary and recurrent fractures in PMOP patients. Lconaru [25] et al. showed that decreased BMD at any site was significantly associated with the occurrence of osteoporotic fractures. The study by Liu [26] and Zhao [27] further identified low BMD as a risk factor for recurrent fractures. The results of the present study were similar to theirs.

Bone turnover markers (BTMs) better reflect bone reconstruction status in the short term than BMD, so monitoring bone resorption and bone formation marker levels in PMOP patients can better prevent fractures risk [28]. The results of this study showed no significant correlation between serum CTX and fractures in PMOP patients. A retrospective study by Fan [4] et al. of 549 postmenopausal women showed that serum CTX was a risk factor for hip fractures in postmenopausal women. The results of our study are inconsistent with them, probably because serum CTX levels are not only influenced by circadian rhythms and food intake but also associated with the underlying diseases of the body [29, 30]. The subjects enrolled in this study were outpatients, the time of testing may not be consistent, and the comorbid diseases are complex, including diabetes, cardio-cerebrovascular diseases, and chronic liver and kidney diseases, etc., which need further observation. This also suggests that patient comorbidities may need to be fully considered when assessing BTMs to predict fractures risk in patients with PMOP. In our study, serum BGP levels were not significantly associated with primary fractures, this result concurred with this of Massera [31] et al. A prospective study by Gui [32] showed that BGP were risk factors for recurrent fractures, which is inconsistent with the results of this study. However, the study was not conducted in patients with PMOP and the sample size for recurrent fractures was small. Parathyroid hormone (PTH) and vitamin D may also influence bone metabolism by regulating calcium and phosphorus homeostasis in the body. Serum Ca and P did not correlate with fractures in PMOP patients in this study, and serum PTH levels were positively correlated with fractures, but the OR was not statistically significant, probably because of the instability of serum biochemical assays. In our study, serum 25 OHD levels did not correlate significantly with fractures in PMOP patients, which is inconsistent with the traditional finding that low 25 OHD levels increase the risk of fractures in the elderly [33]. This may be because serum 25 OHD levels are affected by the season, light, diet and nutritional habits, exercise, medication, etc., and therefore the results may be skewed.

Bone remodeling is regulated not only by bone metabolism but also by other metabolic pathways and factors within the body [34, 35]. Therefore, other comorbidities in patients with PMOP may also contribute to an increased risk of fractures. In our study, after adjusting for confounding factors, regression analysis showed that combining chronic liver and kidney diseases was an independent risk factor for primary fractures in PMOP patients. This result suggested that PMOP patients with chronic liver and kidney diseases are at higher risk of primary fractures. Studies have shown that the pathogenesis of the chronic liver disease is complex and diverse, and the balance of bone reconstruction is affected by the pathogenesis of the chronic liver disease, with different causes of associated bone loss. Mechanisms such as decreased vitamin D and insulin-like growth factor-I (IGF-1), increased inflammatory mediators and fibronectin isoforms can further affect osteoblast and osteoclast function, leading to osteoporosis and an increased risk of fragility fractures [36, 37]. Furthermore, the decreased expression of Kloth protein in chronic kidney disease allows for increased levels of fibroblast growth factor-23 (FGF-23), which in turn leads to decrease calcitriol synthesis and causes secondary hyperparathyroidism; leading to abnormal bone reconstruction. Meanwhile, serum sclerostin, which increases progressively with disease progression, can also inhibit bone formation and bone resorption [38]. This study builds on the previous belief that chronic liver and kidney diseases are associated with fracture and more clearly identifies the effect of combining chronic liver and kidney diseases on the risk of primary fractures in patients with PMOP. In this study, the results also showed a significant positive correlation between the combined cardio-cerebrovascular diseases and primary and recurrent fractures in patients with PMOP. The BMD of the femoral neck and total hip was lower in patients with recurrent fractures in combination with the cardio-cerebrovascular diseases than in patients with recurrent fractures in the absence of cardio-cerebrovascular diseases and patients with primary fractures in combination with the cardio-cerebrovascular diseases. This may be because lipoproteins cause atherosclerosis while also leading to local abnormalities in bone metabolism at end-arterial supply sites such as the femoral neck and hip [39]. It has also been shown that sclerostin can be expressed in the smooth muscle cells of atherosclerotic plaques, thereby inhibiting bone formation [40]. Diabetes was significantly and positively associated with recurrent fractures in PMOP patients in this study. And in the comorbidities subgroup analysis, serum CTX levels were lower in patients with re-fractures with combined diabetes than in patients with re-fractures without combined diabetes and lower than in patients with primary fractures with combined diabetes; serum BGP levels were lower in patients with primary and recurrent fractures with combined diabetes than in patients with primary and recurrent fractures without combined diabetes. This suggests that the rate of bone turnover is reduced in PMOP patients with combined diabetes, which is more obvious in patients with recurrent fractures. This may be because hyperglycemia leads to an increase in advanced glycation end products (AGEs) as well as reactive oxygen species (ROS), which inhibit osteoblast and osteoclast function [41]; Also diabetic patients have higher levels of sclerostin, which may also reduce osteoblast differentiation and proliferation by inhibiting the Wnt/β-catenin signaling pathway [35].

Currently, studies on risk factors for osteoporotic fractures have been reported mainly for primary fractures, with fewer studies on risk factors for postmenopausal recurrent fractures in large samples, and those that have been published have only involved some of the risk factors [42–44]. This study is the first to provide a comprehensive analysis of the clinical characteristics (basic information and comorbidities) and BMD and bone metabolism, etc. as risk factors for recurrent fractures in patients with PMOP, and to compare risk factors for primary and recurrent fractures.

However, there are some limitations to this paper: Firstly, the fractured history of the study subjects was partially self-reported in this retrospective study, which may have biased the information. Secondly, data related to information of fractures on the study population needs to be improved. Thirdly, this study was a single-center study in a Chinese population and the data source was relatively homogeneous. Although we selected 1239 postmenopausal osteoporotic patients with a history of fracture, only 231 of these patients had recurrent fractures. So, the smaller sample size may also have caused some of the differences in indicators not to be better represented. These shortcomings may be supplemented by further multicenter prospective studies with large samples.

## Conclusion

In conclusion, this study shows that advanced age, overweight and low bone mineral density not only increase the risk of primary fractures but also the risk of recurrent fractures in postmenopausal patients with osteoporosis. And combining chronic liver and kidney diseases can also increase the risk of primary fractures in postmenopausal patients with osteoporosis. Therefore, postmenopausal patients with osteoporosis who are elderly, overweight, have low bone mineral density and have chronic liver and kidney diseases need to take early health interventions to reduce the risk of primary and recurrent fractures.

## Data Availability

All data generated or analyzed during this study are included in the article and its additional files.

## Abbreviations

PMOP: Postmenopausal osteoporosis
EPV: Events per variable
DXA: Dual energy x-ray absorptiometry
BMI: Body mass index
CV: Coefficient of variation
OPF: Osteoporotic fracture
BGP: Bone gla protein
CTX: C-termial telopeptide of type 1 collagen
Ca: Calcium
P: Serum phosphorus
PTH: Parathyroid hormone
25 OHD: 25 Hydroxyvitamin D
BMD: Bone mineral density
BTMs: Bone metabolism markers
OR: Odds Ratio
IGF-1: Insulin-like growth factor-I
FGF-23: Fibroblast growth factor-23
AGEs: Advanced glycation end products
